# Seasonal recurrence of cowpox virus outbreaks in captive cheetahs (*Acinonyx jubatus*)

**DOI:** 10.1371/journal.pone.0187089

**Published:** 2017-11-09

**Authors:** Julia Stagegaard, Andreas Kurth, Daniel Stern, Piotr Wojciech Dabrowski, Ann Pocknell, Andreas Nitsche, Livia Schrick

**Affiliations:** 1 Ree Park–Safari, Ebeltoft, Denmark; 2 Centre for Biological Threats and Special Pathogens, Robert Koch Institute, Berlin, Germany; 3 German Consultant Laboratory for Poxviruses, Robert Koch Institute, Berlin, Germany; 4 Methodology and Research Infrastructure, Robert Koch Institute, Berlin, Germany; 5 Finn Pathologists, Norfolk, England; Friedrich-Loeffler-Institut, GERMANY

## Abstract

Cowpox virus infections in captive cheetahs (*Acinonyx jubatus*) with high morbidity and mortality have already been reported in the UK and Russia in the 1970s. However, most of the reported cases have been singular events. Here, we report a total of five cowpox virus outbreaks in cheetahs in the same safari park in Denmark between 2010 and 2014. Nine cheetahs showed varying severity of clinical disease; two of them died (22%). All episodes occurred between August and October of the respective year. No other carnivores kept at the same institution nor the keepers taking care of the animals were clinically affected. The clinical picture of cowpox was confirmed by extensive laboratory investigations including histopathological and molecular analyses as well as cell culture isolation of a cowpox virus. High anti-orthopoxvirus antibody titers were detected in all 9 diseased cheetahs compared to seven contact cheetahs without clinical signs and 13 cheetahs not in direct contact. Additionally, whole genome sequencing from one sample of each cluster with subsequent phylogenetic analysis showed that the viruses from different outbreaks have individual sequences but clearly form a clade distinct from other cowpox viruses. However, the intra-clade distances are still larger than those usually observed within clades of one event. These findings indicate multiple and separate introductions of cowpox virus, probably from wild rodent populations, where the virus keeps circulating naturally and is only sporadically introduced into the cheetahs. Sero-positivity of voles (*Arvicola amphibious*) caught in zoo grounds strengthens this hypothesis. As a consequence, recommendations are given for medical and physical management of diseased cheetahs, for hygienic measures as well as for pre-shipment isolation before cheetah export from zoo grounds.

## Introduction

Cowpox viruses (CPXV) belong to the genus *Orthopoxvirus* (OPV) in the family *Poxviridae* [1]. CPXV are endemic in parts of Europe and Western Asia [2]. The primary reservoir host seems to be rodents [3], as serological surveys have shown that the virus is widespread among European wild rodents [4]. In the wild rodent populations, the virus does not induce obvious signs of disease nor does it seem to affect survival [5]. Despite its name, CPXV has not been isolated from cattle during the last three decades [6]; instead, it has been diagnosed most frequently in domestic cats and zoo animals [7]. After elephants, exotic felids are the second most commonly infected group of exotic animals [8]. Cases in captive cheetahs, with high morbidity and mortality, have already been reported in the UK and Russia in the 1970s [9,10]. In domestic and large cats, multiple skin lesions (primarily seen on the head, neck, forelimb or paws, and/or in the oral cavity), conjunctivitis, and/or purulent ocular discharge may develop upon infection. Occasionally, systemic infections occur that may be fatal if internal organs such as the lungs are involved (e.g. necrotizing pneumonia) and in cases of co-infections or immunodeficiency [11]. The route and site of infection, the dose of virus, as well as the CPXV strain seem to influence the outcome of the infection [12]. Cowpox incidences in cats and humans are reported to be highest in late summer and early autumn [1], which coincides with numbers of seroconverting bank voles (*Clethrionomys glareolus*) and wood mice (*Apodemus sylvaticus*) that peak in this period [3].

**Fig 1:**
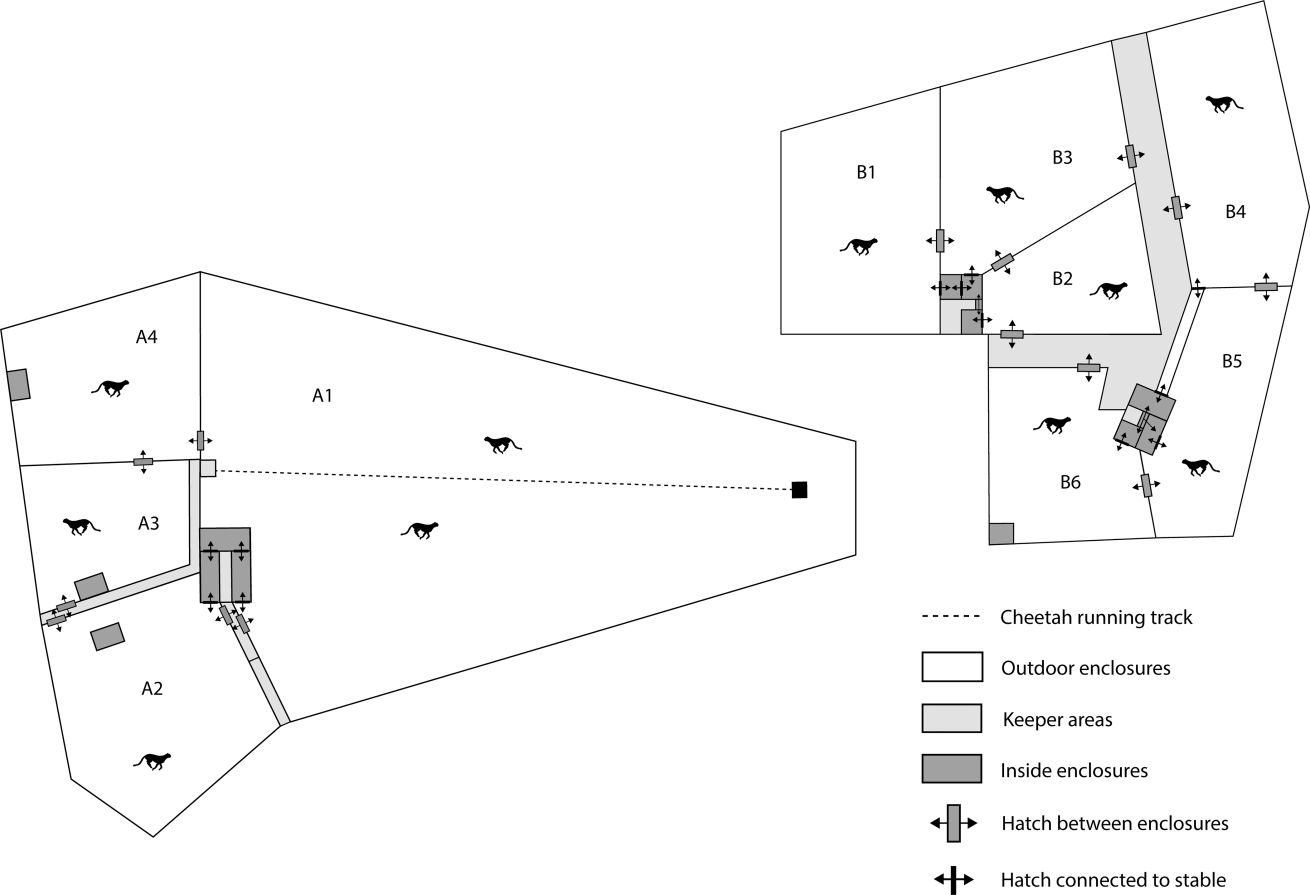
Overview of cheetah enclosures at Ree Park–Safari, Denmark. Enclosures ‘A’ are inside the safari park, enclosures ‘B’ at a farm located 1 km away from the park.

Ree Park–Safari is a 70-ha safari park located on a rural site in the east of Jutland, Denmark. Since 2001, the institution has been keeping cheetahs. The total number of cheetahs kept in the grounds varies; however, usually the zoo houses 2–3 adult males, 3–4 adult females, and their offspring until they are approximately two years old. There are two separate cheetah holding facilities (Fig 1), one in the safari park (Fig 1A) with four pens and one at a farm (Fig 1B) with six pens located 1 km away from the park. All enclosures have natural vegetation with high grass and small bushes. Most of the enclosures connect to a stable varying in size between 4 and 9 m^2^. There is no direct contact between the enclosures as all fences are covered to avoid direct visibility. The same animal keepers take care of both locations.

Beginning with the first outbreak in October 2010, a total of five severe clinical poxvirus infections were diagnosed in cheetahs in the years 2010, 2011, 2012, and 2014. Results of histological and molecular diagnostics, phylogenetic analysis of genome sequences, antibody titers of cheetahs and other animals, as well as management strategies are presented and discussed.

## Materials and methods

### Animal specimens

All organ samples were taken from diseased animals in the context of clinical investigations exclusively for diagnostic purposes or from animals that naturally had died from disease. Therefore no ethics committee has been involved.

None of the animals mentioned in this article was euthanized. All blood samples were drawn during anesthesia (in exotic carnivores at Ree Park always a combination of ketamine and medetomidine, often plus midazolam by remote injection) for either pre-shipment health screening or disease diagnostics. It is state of the art among zoos always to freeze extra blood samples down, either for forensic or for retrospective investigational purposes.

### Histopathology

Samples taken from the lip and the nasal planum, a skin nodule from the index patient in cluster 1, and the toe wound from the index patient in cluster 2 were fixed in 10% neutral buffered formalin and then embedded in paraffin. Sections of the samples were routinely processed, sectioned at 5 μm, and stained with hematoxylin and eosin for histological examination.

### Molecular diagnostics

DNA was extracted from multiple specimens by using the QIAGEN QIAamp DNA Mini Kit. The DNA was analyzed by OPV-specific real-time PCR (rpo 18) [13], CPXV-specific real-time PCR [14], and real-time PCR detecting the cellular *c-myc* gene for relative quantification [15]. Additionally, conventional PCR was performed, amplifying the open reading frame (ORF) of the hemagglutinin (HA) gene followed by sanger sequencing [15].

### Viral genome sequencing and phylogenetic analysis

Full viral genome sequences were gained from one specimen of each cluster by using either DNA from cell culture isolates (if available) or DNA directly isolated from crust or skin tissue. Samples from clusters 1–4 were subjected to IonTorrent PGM sequencing (Ion Xpress Plus Fragment Preparation Kit, Ion PGM™ Sequencing 200 Kit, Ion 318 Chip). For the sample from cluster 5 Illumina sequencing was utilized (Nextera library, TruSeq Rapid PE Cluster Kit v2, HiSeq Rapid SBS Kit v2, 250+250 bases sequencing on a HiSeq 1500 instrument in rapid mode).

Assembly of the sequenced representative specimens’ genomes was performed in three steps. First, reads originating from the host were separated from those originating from the virus by using RAMBO-K [16]. Then genomes were assembled by using a combination of velvet [17] and SOAPdenovo2 [18], yielding the entire viral genome in a single contig of over 200 kb in length in all cases (see S1 Table). Finally, error correction was performed by mapping all reads from each sample to the respective assembled genome by using bowtie2 [19], and discrepancies were visually inspected and, where necessary, corrected manually by using Geneious R9 [20].

For phylogenetic analysis, an alignment of the assembled genomes with all CPXV full genomes and a single representative sequence of every other OPV species available in GenBank was performed by using mafft [21]. In order to remove spurious phylogenetic signals, low-quality alignment regions were stripped from the alignment by using GBlocks [22], yielding a stripped alignment of 136,924 gap-free positions. A phylogenetic tree was then calculated from this stripped alignment by using PhyML [23] (GTR+ɣ, 4 substitution rate categories, aLRT branch supports).

### Virus isolation

Clinical specimens were homogenized in phosphate-buffered saline and added at different dilutions to confluent layers of Vero cells (ECACC, Cat. No 85020206) cultivated with media with 2% fetal calf serum supplemented with penicillin/streptomycin in 24-well cell culture plates. Once cytopathic effects were evident under the microscope, passaging was initiated.

### Serology

Serum samples were tested for antibodies by immunofluorescent assay (IFA) by using CPXV-infected HEp-2 cells and a FITC-conjugated goat-anti-human IgG (H+L) as described previously [24] or by indirect ELISA by using vaccinia virus-infected HEp-2 cell lysate as antigen. To this aim, UV-inactivated cell lysate was prepared by infecting HEp-2 cells (ATCC, CCL-23™) with vaccinia virus strain New York City Board of Health (ATCC, VR-1536™) by using RIPA buffer supplemented with protease inhibitor cocktail (Thermo Fisher Scientific) as described before [25].

For ELISA, PolySorp microwell plates (Nunc) were coated with 100 μL of lysate of infected or non-infected HEp-2 cells at 4 μg/mL in 0.1 M carbonate buffer (pH 9.6) over night at 4°C. Between each step, plates were washed with 4 × 300 μL of washing buffer (100 mM Tris, pH 7.5, 150 mM NaCl, 0.05% Tween 20). Plates were blocked at room temperature for 1 hour with 200 μL per well of 3% bovine serum albumin (BSA, Carl Roth) in washing buffer. Subsequently, 100 μL per well of non-inactivated sera were incubated at a 1:100 dilution in washing buffer supplemented with 0.25% BSA for 1 hour before detection with either goat anti-cat IgG (H+L) HRP, goat anti-dog IgG (H+L) HRP, or goat anti-mouse IgG (H+L) (Dianova, used at a 1:5000 dilution). Finally, signals were developed for 15 minutes by using 100 μL of SeramunSlow TMB substrate (Diavita) per well before the reaction was stopped by addition of 100 μL of 0.25 M H_2_SO_4_. The resulting absorption was read at 450 nm referenced to 620 nm at an ELISA reader (Tecan). Each serum was tested in two replicate measurements on both infected and non-infected HEp-2 cell lysate. Binding signals against non-infected HEp-2 cell lysate were subtracted from signals against vaccinia virus-infected cells, and sera with a differential binding signal above 0.05 were considered positive.

## Results

### Clinical cases

**Fig 2:**
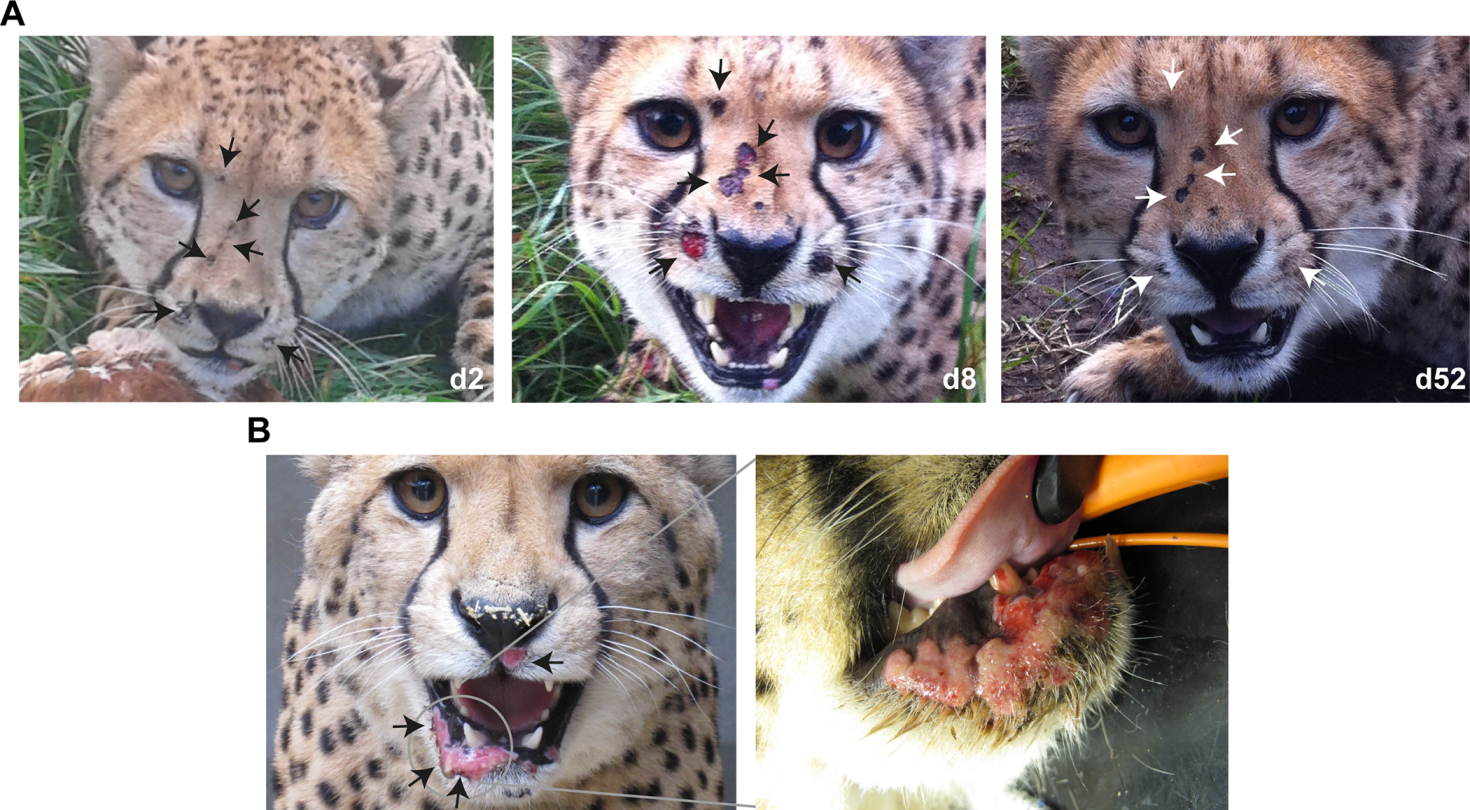
**Exemplary clinical presentations for typical (A) and non-typical (B) poxvirus lesions observed during the outbreaks.** A. Progression of the infection observed for Nova (cluster 5) from d2 when typical dermal nodules were clearly visible, d8 at the peak of clinical disease with multiple classical skin wounds, and d52 with remaining pox scars. Lesions were predominantly located in the face of the animal but were also distributed over the body and legs. B. Atypical ulcerative skin lesions observed on Sheppard (cluster 1). The lesion on the lower lip is shown in the inset on the right for better visibility. (d = days after first clinical signs).

An overview of groups of cheetahs affected in five outbreaks between 2010 and 2014 is given in Table 1, while examples of clinical presentations for typical and atypical lesions observed during the outbreaks are shown in Fig 2.

**Table 1:** Summary of clinical symptoms of the cheetahs affected by five CPXV outbreaks at Ree Park, Denmark.

Cluster	ID	Age	Enclosure	Date of first clinical signs	Clinical signs	Days onset of clinical signs to improvement/ death
1	Sheppard^§^	18 months	A– 1	Oct 4, 2010	Severe facial lesions	12
	Tosha	8 years		Oct 15, 2010	Ulcers on tongue	Unknown
	Grey	18 months		Oct 17, 2010	Lung	12 †
	Izzy	18 months		Oct 18, 2010	Few facial lesions	Unknown
	Split	18 months			-	
2	Top Cut^§^	7 months	B– 1	Aug 31, 2011	Generalized	19 †
	Clyde	7 months		Sep 13, 2011	Ulcers on tongue	Unknown
	Aduke	6 years			-	
	Bonnie	7 months			-	
3	Nuru^§^	7 years	B– 2	Sep 7, 2012	Severe skin lesions	20
4	Hurley^§^	30 months	A– 1	Oct 5, 2012	Facial lesions	13
	Jack	30 months			-	
	Sawyer	30 months			-	
5	Nova^§^	30 months	A– 1	Sep 11, 2014	Severe skin lesions	11
	Heidi	30 months			-	
	Novi	30 months			-	

Listed are the affected animals as well as the contact animals living in the same enclosure but never showing clinical signs. Enclosures: A—situated inside the safari-park, B–situated at a farm 1 km away from the park.

^§^Index case

† death, Age at the time of the outbreak.

#### Cluster 1–2010 (Sheppard)

The first clinical case occurred in the park in October 2010 (Fig 1, enclosure A1), where a mother (Tosha) was housed together with one son (Sheppard) and three daughters (Grey, Izzy, Split). The index patient was the 1.5-year-old son Sheppard. He presented with ulcerative skin lesions on the lower lip and nostril. The affected areas were biopsied and the animal received long-acting broad-spectrum antibiotic (8 mg/kg cefovecin once i.m.). The animal was isolated in the stable ten days after initial symptoms when histopathological results of the submitted samples indicated presumptive poxvirus infection.

Eleven days after the first lesions had been noticed in the male, a fissure was observed on the tongue of the mother (Tosha), and on the following day one of its sisters (Izzy) had a lesion on the upper lip. The next day, respiratory distress became obvious in another sister (Grey). At that time, the entire group was isolated and put inside the stable. Cefovecin was given by remote injection to all of them in order to prevent secondary bacterial infections.

The female Grey with respiratory symptoms died 12 days after onset of symptoms and was found partly eaten by the others. The other animals recovered uneventfully. Only one of the five animals (Split) did not show any symptoms.

#### Cluster 2–2011 (Top Cut)

After the first outbreak in the park, the second one occurred at the farm (Fig 1, enclosure B1) in August 2011, in a family group consisting of a breeding female (Aduke) and two male (Top Cut, Clyde) and one female offspring (Bonnie). The index patient, a 7-month-old male (Top Cut), presented with a poorly healing wound on the front paw. Upon clinical examination of the cheetah under general anesthesia, multiple papular dermal nodules were noticed on the entire body, and poxvirus infection was suspected. Despite antibiotic and anti-inflammatory treatment (initially 8 mg/kg cefovecin i.m. once, from day 2 clindamycin 11 mg/kg BID p.o., and meloxicam 0.05 mg/kg SID p.o.), the animal died 19 days after the disease was first noticed.

Only one of its two siblings (Clyde) developed an ulcer on the tongue on day 14, the other sibling (Bonnie) and the dam did not show any signs of infection at all. The animals remained together in their enclosure throughout the disease process.

#### Cluster 3 – 2012a (Nuru)

In September 2012, a 7-year-old male (Nuru), housed alone at the farm in the neighboring enclosure to cluster 2 (Fig 1, enclosure B2), developed severe pox-like lesions in the face. Within days, the entire body was covered by visible pustules. To avoid secondary infections and to reduce scratching, the animal was kept under oral antibiotics (5 mg/kg enrofloxacin SID p.o.) and non-steroidal anti-inflammatories (0.05 mg/kg meloxicam SID p.o.) until obvious improvement on day 20. He recovered uneventfully and was not isolated during the period.

#### Cluster 4 – 2012b (Hurley)

One month later, in October 2012, a 2.5-year-old male (Hurley), housed at the park (Fig 1, enclosure A1) together with his two brothers (Jack, Sawyer), had typical pox lesions only in the face. He recovered after 13 days without any treatment. No lesions were seen in the siblings.

#### Cluster 5–2014 (Nova)

Starting in 2013, all cheetahs were vaccinated with Modified Vaccinia Ankara (MVA) smallpox vaccine, following the vaccination regime as recommended by the producer. However, another outbreak occurred in September 2014 in the park (enclosure A1) where three female siblings were living (Nova, Novi, Heidi). The 2.5-year-old Nova showed severe skin lesions consistent with cowpox in the face, on her body, and her legs. She received daily oral antibiotics (5 mg/kg enrofloxacin SID p.o.) and improved after 11 days. Her two sisters did not show any lesions.

### Histopathology

Sections from the lip and nasal planum of Sheppard (cluster 1) showed diffuse ulcerative exudative necrotic cheilitis and nasal dermatitis, respectively–the ulcer on the nasal planum was superficially colonized by mixed bacteria. Sections from the skin nodule showed similar ulceration, exudation, and necrosis, but also showed some areas with intact epidermis and hair follicles. Keratinocytes in the epidermis and in some hair follicle walls showed extensive disruption and hydropic degeneration, often accompanied by one to several large, angular, eosinophilic, cytoplasmic inclusion bodies. The combination of the necrotic and ulcerative inflammation and the cytoplasmic epithelial inclusion bodies led to a presumptive diagnosis of cutaneous poxviral infection, with suspicion of CPXV infection. Sections of the skin at the site of the toe wound from Top Cut (cluster 2) showed very similar histological lesions, comprising ulcerative necrotic dermatitis, folliculitis, and perifolliculitis, again with similar cytoplasmic eosinophilic inclusion bodies in keratinocytes, supporting the presumptive diagnosis above.

### Molecular diagnostics

Samples from all index cases (Sheppard, Top Cut, Nuru, Hurley, Nova) and the dead female of cluster 1 (Grey) were submitted to the German Consultant Laboratory of Poxviruses at the Robert Koch Institute for diagnostics and further classification of the viruses. PCR analysis showed high OPV-DNA loads when normalized to cellular DNA (*c-myc*) in multiple specimens from several affected animals (Table 2). Viral load was especially high in the deceased animals (Grey and Top Cut) with extraordinarily high viral loads detected in the blood, indicating a systemic infection.

**Table 2:** Real-time PCR results of different specimens from symptomatic cheetahs at Ree Park.

Cluster	ID	Time from first symptoms to sampling in d	Material	Cq OPV	Cq c-myc	ΔCq (OPV— c-myc)
1	Sheppard^§^	3	Skin nodule (lip, formalin-fixed)	28.2	34.2	-6.0
	Grey†	12	Lung tissue*#	9.8	20.7	-10.9
		12	Blood	19.9^&^	-	-
		12	Vulvar swab	29.9	21.6	8.3
		12	Oral swab	24.0	23.1	0.9
		12	Rectal swab	29.4	22.3	7.1
2	Top Cut^§^†	19	Skin tissue*	15.0	21.3	-6.3
			Oral swab	24.3	26.2	-1.9
			Rectal swab	19.8	23.7	-3.9
			Preputial swab	15.7	22.6	-6.9
			Body fluids abdominal cavity	25.0^&^	-	-
			Lung tissue	23.4	24.7	-1.3
			Blood	17.8^&^	-	-
3	Nuru^§^	11	Crust*#	13.1	20.5	-7.4
4	Hurley^§^	7	Crust*#	20.7	20.5	0.2
5	Nova^§^	18	Crust*	17.8	20.5	-2.7

Symptomatic animals from each cluster were sampled for PCR analysis. Multiple specimens were collected from the deceased animals Grey and Top Cut. For each sample OPV DNA (located in the rpo18 gene) was quantified in relation to cellular c-myc DNA. Lower values for ΔCq indicate higher virus loads in a respective tissue. Cq, quantification cycle; OPV, orthopoxvirus

^§^ index case

†death

^&^Cq value per 5 μl of DNA

#material used to obtain cell culture isolate

*material applied to CPXV-specific real-time PCR, HA-sequencing, and next-generation sequencing.

For each cluster, selected samples were analyzed further as indicated in Table 2. A CPXV-specific real-time PCR and HA sequencing confirmed the infection with OPV and further classified the causative agent as CPXV. Virus was isolated from cell culture for samples indicated in Table 2.

#### Viral genome sequencing and phylogenetic analysis

**Fig 3:**
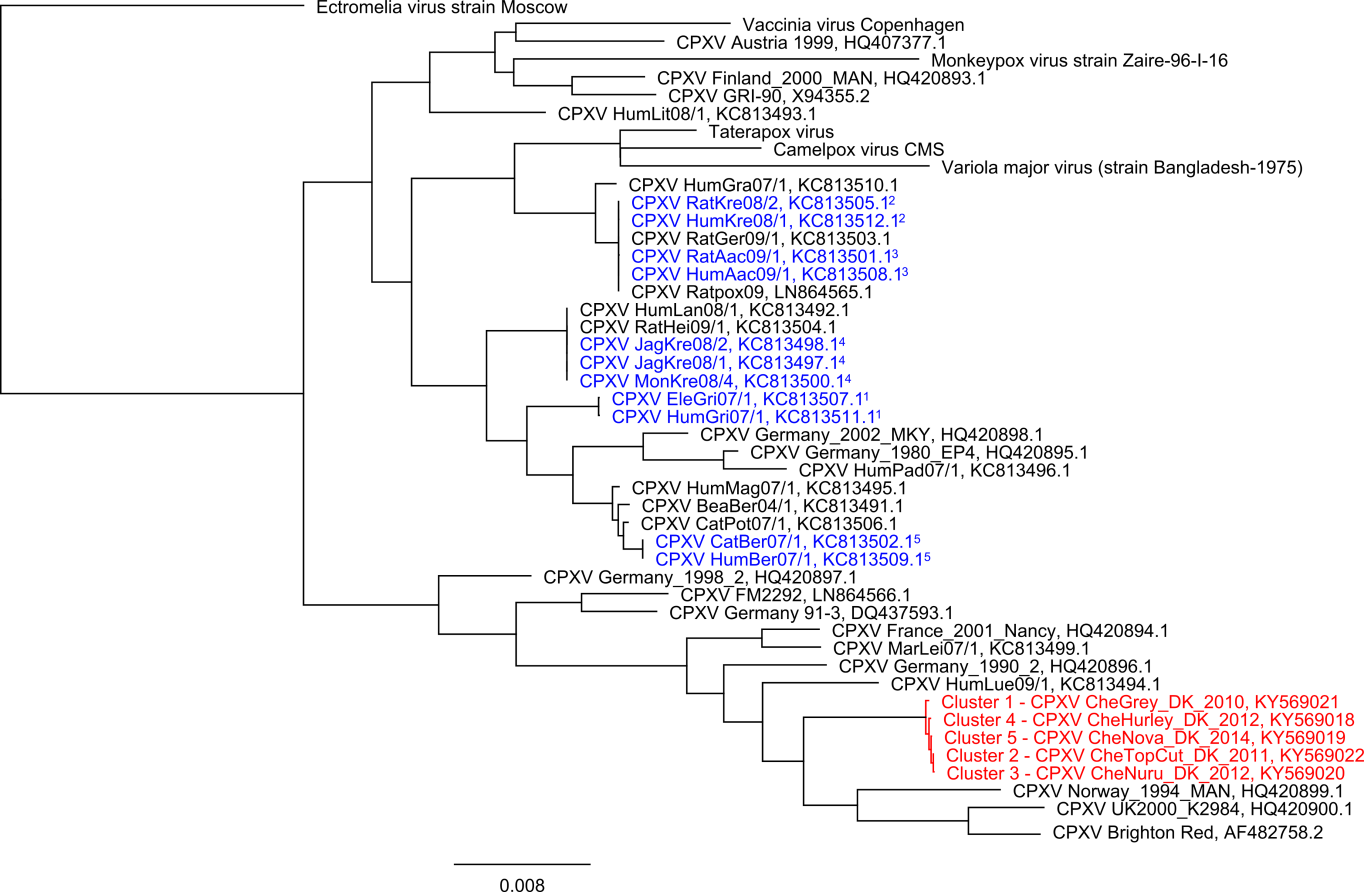
Phylogenetic placement of CPXV from outbreak clusters in cheetahs among all known CPXV genomes and one representative sequence from every other OPV species. Maximum likelihood tree based on stripped whole-genome alignment. The representative strains of the CPXV clusters from cheetah are highlighted in red. Groups of CPXV strains belonging to known outbreaks are colored in blue, same superscripts denote the same outbreak. The clustering between the sequences from the known outbreaks (blue) is much tighter than between the sequences from the outbreaks in cheetah (red). All branch supports (aLRT [26]) are above 0.99.

Furthermore, a whole genome sequence was reconstructed from one sample of each cluster (for selection of material see Table 2, for contig length and accessions see S1 Table), and phylogenetic analysis was performed. In the resulting tree (see Fig 3), the five representative outbreak sequences from Ree Park clearly form a distinct clade, with intra-clade distances much lower than the distance of any of the outbreak sequences to any other known CPXV. However, the intra-clade distances are still larger than those observed within clades formed by other CPXV known to come from distinct outbreak events.

#### Serology

All serological results of blood samples from cheetahs obtained during or shortly after the outbreaks as well as results from follow-ups and cheetahs within the institution that have never shown any symptoms of a poxvirus infection are collected in Table 3. All symptomatic animals developed high OPV-antibody titers within the first month after symptom onset with IgM≥1:320 and IgG≥1:20,480. The IgM titer was already clearly elevated in the two sera taken from Sheppard and Top Cut 3 days after symptom onset and decreased within the course of infection. On the other hand, animals that were kept together in the stable or enclosure with the symptomatic animals but remained symptom-free developed only moderate antibody titers, indicating asymptomatic and mild infections. The animals that had never been in contact with symptomatic animals showed no or only low OPV-antibody responses (IgM≤80 and IgG≤1:320), whereas in our experience only IgM titers ≥ 80 and IgG titers > 320 are considered meaningful.

**Table 3:** Anti-OPV antibody titers of cheetahs at ReePark.

Cluster	ID	Date of blood sampling	Time from first symptoms to sampling	IgM	IgG
1	Sheppard^§^	Oct 7, 2010	3 days	≥1:5,120	1:20,480
	Grey†	Oct 29, 2010	12 days	1:320	1:81,920
	Izzy	Dec 5, 2010	2 months	1:80	1:81,920
	Split	Dec 6, 2010		1:80	1:20,480
2	Top Cut^§^	Sep 2, 2011	2 days	1:5,120	1:20,480
	Top Cut^§^†	Sep 19, 2011	19 days	1:1,280	1:20,480
	Bonnie	Jun 17, 2015		1:20	1:1,280
3	Nuru^§^	Jun 29, 2014	22 months	1:80	1:20,480
4	Hurley^§^	Oct 12, 2012	7 days	1:1,280	1:81,920
	Hurley^§^	Oct 7, 2013	12 months	1:20	1:5,120
	Sawyer^&^	Jul 15, 2014		1:20	1:1,280
	Jack^&^	Jul 15, 2014		1:80	1:1,280
5	Nova^§^^&^	Sep 29, 2014	18 days	1:320	1:20,480
	Nova^§^^&^	Dec 10, 2014	3 months	1:20	1:20,480
	Novi^&^	Dec 10, 2014		<1:20	1:1,280
	Heidi^&^	Dec 10, 2014		1:20	1:1,280
control animals	Yellow Eye	Jul 2, 2007		<1:20	1:80
	Desert	Sep 9, 2011		<1:20	1:20
	Cimber	Mar 7, 2012		< 1:20	1:320
	Sterling	Mar 7, 2012		< 1:20	1:20
	Suna	Jul 10, 2012		1:20	1:80
	Duma	May 22, 2013		< 1:20	1:80
	Hollaender^&^	Jun 28, 2014		1:80	1:320
	Abayomi^&^	Jan 28, 2015		<1:20	1:320
	Maya^&^	Nov 2, 2015		<1:20	1:320
	Sarah^&^	Nov 2, 2015		<1:20	1:320
control animals*	Tosha*	Sep 14, 2010		< 1:20	1:80
	Jack*	Feb 21, 2011		<1:20	1:80
	Sawyer*	Feb 21, 2011		1:20	1:320
	Kate*	Sep 5, 2012		< 1:20	1:80

Anti-OPV antibody titers were determined by Immunofluorescence assay (IFA). In the upper part of the table (clusters 1–5) results from CPXV-infected individuals are shown with indication of timespan from symptom onset to sampling. These affected animals developed high OPV antibody titers. Furthermore, titers from animals that shared the same enclosure but remained symptom free are shown in the upper part of the table. These animals show only moderate antibody responses. Animals that had never been in direct contact with an affected animal and thus are considered as control animals developed no or only low antibody responses. These results are shown in the lower part. In our experience, only IgM titers ≥ 80 and IgG titers > 320 are considered meaningful.

^§^Index case

†death

*samples from animals involved in clusters 1–5 taken before the outbreak

^&^previously vaccinated with MVA.

Additionally, other animals kept or caught in the zoo were analyzed for OPV antibody status. Therefore, a new ELISA was established which showed excellent agreement with IFA data (see S1 Fig). An overview of seroreactivity in felines other than cheetahs kept in the zoo, as well as voles and other animals caught in zoo grounds, is given in Table 4. Although none of these animals showed symptoms compatible with CPXV infection, OPV seropositivity was shown for multiple animals from different species. In contrast to the strong seroreactivity observed in symptomatic cheetahs, here the overall antibody reactivity against OPV as tested by IFA and ELISA was low (maximum IFA titers of 1:1,280). Additionally, sera from five animal keepers taking care of the cheetahs were analyzed for OPV-antibodies within months of the outbreaks (data not shown). None of the keepers developed symptoms and they showed negative or only weakly positive OPV-antibody titers by IFA, indicating no severe acute infection.

**Table 4:** OPV seropositivity of other animals kept at the institution or caught in zoo grounds between 2011 and 2015.

Kept at the institution (Inst)/Wild caught (Wild)	Species	No of individuals tested	No of seropositive animals
Inst	Lion (*Panthera leo*)	13	3
Inst	Sumatran tiger (*Panthera tigris sumatrae*)	1	1
Inst	Leopard (*Panthera pardus*)	1	0
Inst	Sand cat (*Felis margarita*)	2	0
Wild	Domestic cat (*Felis catus*)	1	1
Wild	Red fox (*Vulpes vulpes*)	1	1
Wild	Water vole (*Arvicola amphibius*)	21	14

Serostatus was determined by IFA and/or ELISA, whereas an IgG ≥ 1:320 or differential ELISA > 0.05 is considered positive. Anti-OPV antibodies were detected in multiple animals from different species.

## Discussion

Cheetahs seem to be more prone to CPXV infections than other exotic cat species [9,10,27]. During an outbreak at Whipsnade Park in the UK in 1977, two out of three symptomatic cheetahs died; another six contact animals did not have any clinical infection [9]. In contrast, during the outbreak at Moscow Zoo, Russia, in 1973 [10], many different feline species, including two cheetahs, all housed in the same building, fell ill and the majority died. In a recent outbreak at Chester Zoo, UK, in 2012, a family group of five cheetahs got sick and two 4-month-old cubs died, but four other cheetahs in the same institution were not affected [27]. Even though other carnivores were also kept at Whipsnade and Chester, only cheetahs developed clinical poxvirus infection. In both events, only contact animals became sick, indicating high-quality hygiene standards preventing transmission through staff. These observations are in accordance with the outbreaks at Ree Park: although many other feline species (see Table 4) are kept under similar conditions, in close proximity to the cheetahs, and are looked after by the same keepers, none of the animals or keepers has ever shown any symptoms. The reason of cumulated CPXV infections of cheetahs compared to other animal species might be a reduction of genetic diversity, correlating with an increased vulnerability to infectious diseases [28], similar to their high mortality after feline infectious peritonitis (FIP) infections which has been reported previously [29].

The majority of the reported cases from other zoos have been singular events. Yearly recurrence as in Ree Park has not been described to such an extent earlier. Pfeffer [30] has previously suggested that the cause of sporadic CPXV infections in domestic cats is probably related to the prevalence of CPXV in local rodent populations. Due to the availability of food all the year round and because of the dense vegetation, high rodent activity in and around the zoo is always present. Even though no CPXV could be isolated, 14 out of 21 examined water voles (*Arvicola amphibius*) caught in the grounds showed antibody titers against poxvirus (Table 4). Also, the fact that outbreaks occurred over a 5-year period, always during late summer/autumn (August–October), is in agreement with other authors [1] and is another indication that poxvirus was not introduced through food animals but rather that wild rodents may have been the source of infection.

The route of infection in the index patients cannot be proven; however, in most cheetahs, the first signs were either at the mouth or on the paws. A possible explanation for this distribution of lesions is that cheetahs actively catch rodents and get infected following injury caused by their prey. Furthermore, domestic cat-to-cat transmission is described in the literature as a rare event [11]. Nevertheless, in our cases, we favor the idea of subsequent cheetah-to-cheetah transmission over multiple rodent-to-cheetah transmissions within one cluster since symptoms were first observed 11–12 days after the respective index patient became sick. Although symptoms in contact animals were only observed in two of the four enclosures housing more than one animal, high OPV-antibody titers in animals in contact with diseased animals (Table 3) indicate further spread and inapparent infections.

Even though OPV have a high tenacity in the environment [31], it is unlikely that the three clusters in enclosure A1 were caused by identical virus material from the environment. All five isolates varied to some extent in the genome sequence (Fig 3), indicating that they evolved and were introduced multiple times from their reservoir hosts. Also, the two CPXV that were found in the farm and park within four weeks (clusters 3 and 4) clearly differ in their sequences, indicating the co-circulation of multiple viruses. Furthermore, we conclude that the virus was most likely not keeper transmitted. While the sequences from the five cheetah clusters do not cluster as closely as other sequences coming from a single outbreak (see Fig 3), this is not surprising given that the examined events span several years. The low intra-clade distances and the comparatively large distance to any sequence from outside the cheetah clade are also consistent with the hypothesis of CPXV circulating and evolving in the wildlife in the region and sometimes becoming visible due to transmission to cheetahs in the zoo.

Ongoing serological tests of other felines kept in the zoo revealed seropositivity also in other felines such as a Sumatran tiger (*Panthera tigris sumatrae*) and lions (*Panthera leo*) (Table 4). Despite close observation, no clinical disease was noticed in any species other than cheetahs. The route of infection in these animals is unknown, but it seems most likely that these other felines also acquired the virus directly from rodents. Future research would be needed to see if these species develop clinical disease under certain circumstances (e.g. stress, immunosuppression, or high dose of virus exposure).

Clinical signs varied among the different cases. Since none of the cheetahs at Ree Park has ever shown severe symptoms for or succumbed to any common cat virus, it is unlikely that such a virus has promoted clinical manifestation of CPXV disease. However, apart from the female that died with severe respiratory symptoms, it was always the index patient that was clinically most severely affected. The detection of high viral loads in multiple tissues and especially in the blood of Grey and Top Cut (Table 2) is consistent with the observed severity of disease. Contact animals only developed mild lesions which were only noticed because of the careful monitoring during outbreaks. These observations suggest an inefficient transmission of the virus from cheetah to cheetah which is in concordance with the description of rare events of cat-to-cat transmission [11]. As already stated by others, the route and site of infection as well as the dose of virus seem to influence the clinical outcome [12]. It is not known whether the low mortality at Ree Park in comparison to the mortality in other published outbreaks is due to a CPXV strain with low virulence or whether different management during the outbreaks contributed to a higher survival rate. During the first outbreak (cluster 1), the entire group of five animals was isolated in their stable, resulting in a very high stress level. The siblings of this cluster (Table 3, cluster 1) were the contact animals with the highest post-infection antibody titers at the zoo, which might be a result of greater exposure to virus material inside the environment of the stable or of multiple infection events with high viral loads as they had partly eaten their dead sibling. After this first incident, animals with pox lesions were not confined to a stable or handled, in order to keep stress levels as low as possible. Also, the vegetation was kept untouched to increase the wellbeing of the cheetahs, rather than trying to reduce the number of rodents. In current literature [8,32], isolation is considered the most important management tool to avoid further spread of the disease. In our experience, the virus is not easily spread from one enclosure to another. Because the enclosures at Ree Park are large, keepers only need to enter the enclosures once to twice a year for maintenance reasons. Whenever any carnivore enclosure is entered, a double layer of single-use disposable overshoes is worn to avoid transmission of pathogens from one area to another. Single-use disposable gloves are always worn when animals or their food or waste are handled. The same standards are used for entering stables or night houses. By leaving the animals outdoors and sticking to the hands-off policy by the keepers, the risk of zoonotic infections for the keepers is reduced as well.

In recent years, several antiviral drugs have been developed for the pet industry, but data on its use in exotic cats is limited. Due to the hands-off policy during poxvirus infection, no antiviral drug has been used yet.

Because the outbreaks recurred on a yearly basis, all cheetahs were vaccinated in 2013 by using the Modified Vaccinia Ankara (MVA) smallpox vaccine which is widely used in elephants for prevention of clinical disease [33]. The same vaccination has already been used also in felids, but only sporadically, and information of antibody responses is limited [24]. All cheetahs were initially vaccinated with 2ml MVA twice, with a 4-week interval. Although cluster 5 in 2014 was a group of vaccinated animals, one animal (Nova) was still infected with CPXV. This implies that the affected animal was not completely protected from disease, indicating that cheetahs might need a different vaccination regimen to that recommended in elephants. However, it is possible that the vaccination might have helped to prevent a more severe course of disease in Nova and infection of the siblings.

With almost yearly poxvirus outbreaks in the cheetahs since 2011, the area is currently considered endemic for CPXV. As the morbidity and mortality are rather low compared to those in other reports, the only applied husbandry change has been basic MVA vaccination of cheetahs born in the zoo (2 ml twice with 4-week interval, first vaccination at an age of 8 weeks for handling reasons) and yearly booster vaccinations of all adult cheetahs (2 ml once). Animals other than cheetah are currently not vaccinated at the institution. Modification of enclosure design, increased pest control, and aggressive treatment in the case of CPXV infections have not been applied. However, as vaccination has not prevented clinical infection, a 4-week preshipment quarantine for all cheetahs that leave the institution has been made obligatory. The quarantined animals are kept inside a stable from the day a first blood sample is taken until the day they leave. After four weeks, a second blood sample is drawn. The samples are tested for OPV antibodies by IFA. No change or a decrease in antibodies is considered to be negative for signs of current infection. To date, preshipment quarantine at Ree Park is only carried out in cheetahs. Whether the preshipment quarantine should be extended to other species remains the subject of discussion.

Even though occupational risk for poxvirus infections has been reported for animal keepers [34], it seems unlikely that keepers or visitors could contract the disease at Ree Park, as there is no direct contact between visitors and the cheetahs, and as the above-mentioned hygienic measures are always applied when keepers get in contact with animals or enter the stables and enclosures. The fact that neither the veterinarian nor any of the carnivore keepers have developed symptoms compatible with CPXV or high antibody titers against OPV since the first outbreak supports this opinion.

## Supporting information

S1 Table Results of the full genome assembly of sequenced strains. (DOCX)

S1 Fig Correlation between IFA titers and ELISA results for a subpanel of n = 12 cheetah sera. To determine the agreement between the newly established anti cat IgG ELISA and the well-established IFA, a small subpanel of sera was analysed by both methods. Log-transformed IFA titers and ELISA readings showed highly concordant results. Slashed red line: cutoff value of 0.05 to discern positive from negative sera. Overall, a high degree of correlation between both methods (Pearson r = 0.97) was found.
(TIF)
