# Supplementary material for: Seasonal recurrence of cowpox virus outbreaks in captive cheetahs (Acinonyx jubatus)
Source: PLoS One. 2017 Nov 9;12(11):e0187089. doi: 10.1371/journal.pone.0187089 (PMC5679633; doi:10.1371/journal.pone.0187089)
Supplement: S1 Table — (DOCX) [file pone.0187089.s001.docx]

**S1 Table. Results of the full genome assembly of sequenced strains.**

| Name | Contig length | GenBank accession | SRA accession |
| --- | --- | --- | --- |
| CPXV CheGrey_DK_2010 | 204321 | KY569021 | SAMN07518965 |
| CPXV CheTopCut_DK_2011 | 204015 | KY569022 | SAMN07518966 |
| CPXV CheNuru_DK_2012 | 203925 | KY569020 | SAMN07518967 |
| CPXV CheHurley_DK_2012 | 204218 | KY569018 | SAMN07518968 |
| CPXV CheNova_DK_2014‎ | 214869 | KY569019 | SAMN07518969 |
