# Supplementary figures and images for: Seasonal recurrence of cowpox virus outbreaks in captive cheetahs (Acinonyx jubatus)
Source: PLoS One. 2017 Nov 9;12(11):e0187089. doi: 10.1371/journal.pone.0187089 (PMC5679633; doi:10.1371/journal.pone.0187089)

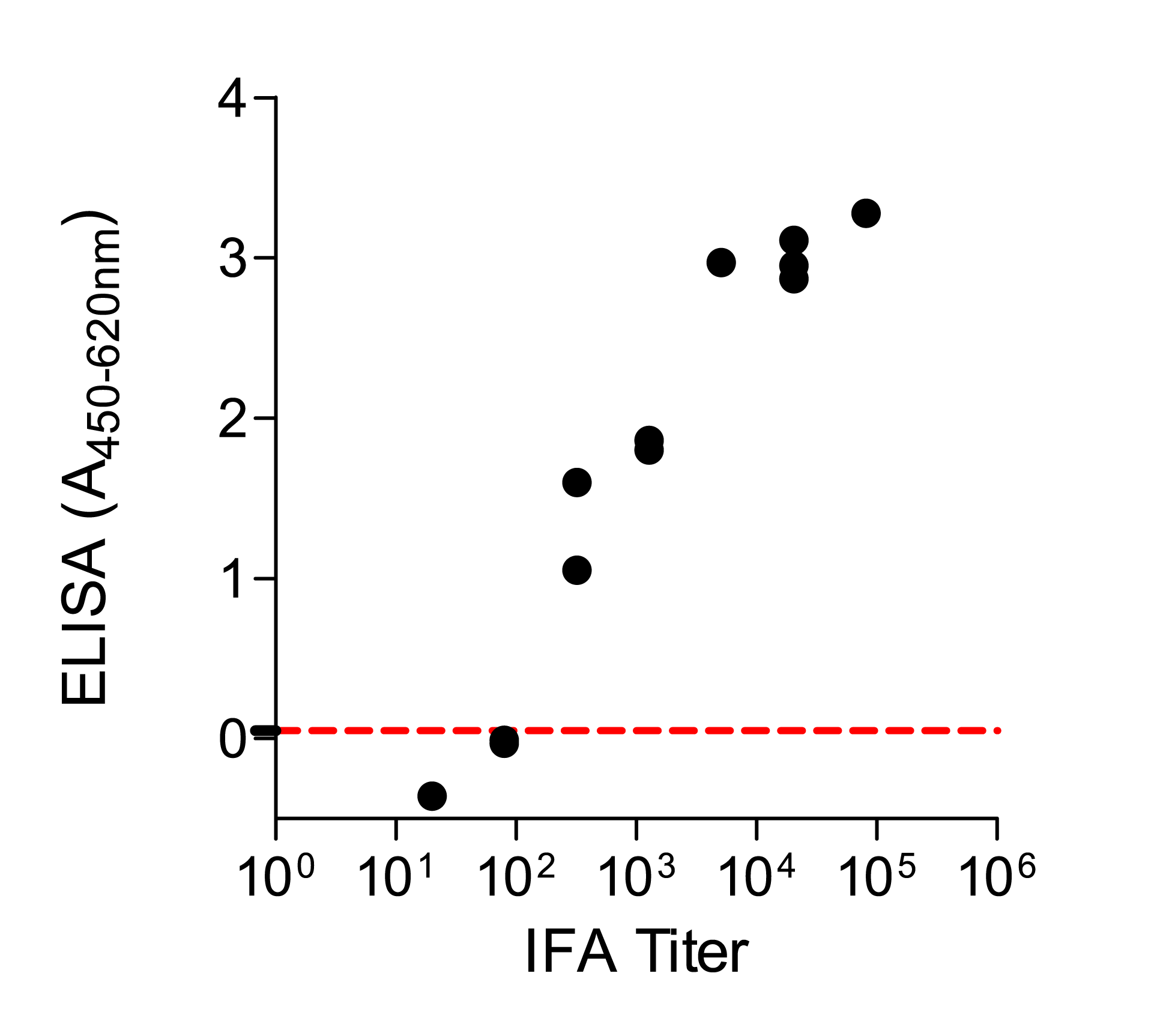

Supplement: S1 Fig — To determine the agreement between the newly established anti cat IgG ELISA and the well-established IFA, a small subpanel of sera was analysed by both methods. Log-transformed IFA titers and ELISA readings showed highly concordant results. Slashed red line: cutoff value of 0.05 to discern positive from negative sera. Overall, a high degree of correlation between both methods (Pearson r = 0.97) was found. (TIF) [file pone.0187089.s002.tif]
